# New Insights into Genetics of Endometriosis—A Comprehensive Literature Review

**DOI:** 10.3390/diagnostics13132265

**Published:** 2023-07-04

**Authors:** Diana Maria Chiorean, Melinda-Ildiko Mitranovici, Havva Serap Toru, Titiana Cornelia Cotoi, Alexandru Nicușor Tomuț, Sabin Gligore Turdean, Ovidiu Simion Cotoi

**Affiliations:** 1 Department of Pathology, County Clinical Hospital of Targu Mures, 540072 Targu Mures, Romania; sabiturdean@yahoo.com (S.G.T.); ovidiu.cotoi@umfst.ro (O.S.C.); 2 Department of Obstetrics and Gynecology, Emergency County Hospital Hunedoara, 14 Victoriei Street, 331057 Hunedoara, Romania; mitranovicimelinda@yahoo.ro; 3 Department of Pathology, Akdeniz University School of Medicine, Antalya Pinarbasi Konyaalti, 07070 Antalya, Turkey; seraptoru@akdeniz.edu.tr; 4 Department of Pharmaceutical Technology, “George Emil Palade” University of Medicine, Pharmacy, Sciences and Technology, 540142 Targu Mures, Romania; titiana.cotoi@umfst.ro; 5 Faculty of Medicine, “George Emil Palade” University of Medicine, Pharmacy, Sciences and Technology, 540142 Targu Mures, Romania; nicusortomut19@gmail.com; 6 Department of Pathophysiology, “George Emil Palade” University of Medicine, Pharmacy, Science, and Technology of Targu Mures, 38 Gheorghe Marinescu Street, 540142 Targu Mures, Romania

**Keywords:** endometriosis, genetics, diagnostic techniques, genetic markers, molecular pathways, genetic heterogeneity, multi-omics, personalized management

## Abstract

This comprehensive review explores the genetic contributions to endometriosis and their potential impact on improving diagnostic techniques. The review begins by defining endometriosis and discussing its prevalence, emphasizing the need for a deeper understanding of the genetic basis of the condition. It highlights recent genome-wide association studies (GWAS) that have identified specific genetic variants associated with endometriosis, shedding light on the molecular pathways and mechanisms involved. The review addresses genetic heterogeneity across different populations and ethnicities, emphasizing the importance of considering population-specific markers in diagnostic approaches. It explores the diagnostic implications of genetic insights, including the potential use of genetic markers for precise and early detection, as well as risk prediction. The review also delves into the integration of genetic information with clinical parameters and imaging findings, and the exploration of multi-omics approaches for a comprehensive understanding of endometriosis. It discusses recent studies on genetic and epigenetic biomarkers, their potential as diagnostic tools, and the need for validation in independent cohorts. The review highlights the impact of new genomic technologies, such as next-generation sequencing, in improving diagnostic accuracy and personalized management. It identifies the challenges and future directions in translating genetic findings into diagnostic tools and emphasizes the transformative potential of genetic insights in endometriosis diagnosis. This review provides a roadmap for future research and underscores the significance of genetic insights in improving diagnostic precision and personalized care for individuals with endometriosis.

## 1. Introduction

Endometriosis, a prevalent yet often misunderstood gynecological condition, affects approximately 10% of women globally during their reproductive years, exerting a substantial toll on their lives [1]. The condition involves the abnormal growth of endometrial-like tissue outside the uterus, leading to a complex and multifactorial disorder that impacts various aspects of a woman’s life. Beyond the physical manifestations, endometriosis adversely affects mental health, work productivity, relationships, and overall quality of life [2,3].

Despite its high prevalence and profound impact, the underlying mechanisms of endometriosis remain elusive, and reliable non-invasive diagnostic methods are lacking. Currently, invasive surgical procedures like laparoscopy coupled with histological confirmation are the gold standard for diagnosis. However, the absence of specific non-invasive diagnostic tools for endometriosis and the heterogeneous clinical presentation of the disease contribute to delayed diagnosis or misdiagnosis, with an average delay of 7–10 years from symptom onset to definitive diagnosis. Such delays can worsen disease progression, intensify symptoms, and negatively impact fertility. Consequently, there is an urgent need for improved diagnostic approaches [1,3,4].

It is important to note that CA125, also known as cancer antigen 125, can provide valuable information, but it is not a definitive diagnostic tool for endometriosis. The test has limitations, as CA125 levels can be influenced by other factors, such as menstrual cycle phase, other gynecological conditions (ovarian cysts, pelvic inflammatory disease), and even non-gynecological conditions (liver disease). Therefore, CA125 should be interpreted in conjunction with clinical evaluation, imaging studies, and other diagnostic tests.

Further research and clinical guidelines are needed to fully establish the role of CA125 in the diagnosis and management of endometriosis [5].

Understanding the genetic contributions to endometriosis has emerged as a promising avenue for enhancing diagnosis. Familial aggregation and twin studies have provided compelling evidence of a strong heritable component in endometriosis. The increased risk of developing the condition among first-degree relatives of affected women indicates a pivotal role for genetic factors in its pathogenesis.

Advancements in genomic technologies have facilitated the exploration of the genetic complexities underlying endometriosis. Genome-wide association studies (GWAS) and next-generation sequencing have identified numerous genetic loci and variants associated with endometriosis, offering insights into potential molecular mechanisms and paving the way for genetic risk prediction models and non-invasive biomarker discovery.

The integration of genetic insights into the diagnostic toolkit has the potential to revolutionize the approach to endometriosis. It envisions a future where early and accurate diagnosis enables timely interventions and personalized treatment strategies. The genetic stratification of patients could lead to targeted therapies that improve outcomes and enhance quality of life [2].

In this comprehensive review, we delve into the captivating realm of endometriosis genetics, focusing on the transformative potential of genetic discoveries in revolutionizing the diagnosis of this enigmatic disorder. By understanding the genetic underpinnings, we aim to pave the way for improved diagnostic techniques, personalized treatment approaches, and, ultimately, better outcomes for individuals with endometriosis [2,3,4,5].

## 2. Material and Methods

A comprehensive literature search was conducted across PubMed databases. The search typically involved specific keywords and phrases such as “endometriosis,” “genetics,” “genetic variants,” “diagnosis,” “biomarkers,” and “genome-wide association studies.” The search was typically filtered to include articles published within a specific timeframe, between 2013 and 2023.

The titles and abstracts of the identified articles were screened to assess their relevance to the topic. The full texts of 150 potentially relevant articles were accessed and scrutinized to ensure they meet the inclusion criteria for the review. Studies were included if they provided original data on the genetic underpinnings of endometriosis and discussed potential diagnostic implications. Any non-English articles were excluded. The findings were interpreted in the context of the current understanding of endometriosis and its genetic basis. 

### PubMed Search Strategy

182 results

(“endometriosis”[MeSH Terms] OR “endometriosis”[All Fields] OR “endometrioses”[All Fields]) AND (“genetic”[All Fields] OR “genetical”[All Fields] OR “genetically”[All Fields] OR “genetics”[MeSH Subheading] OR “genetics”[All Fields] OR “genetics”[MeSH Terms]) AND (“genetic”[All Fields] OR “genetical”[All Fields] OR “genetically”[All Fields] OR “genetics”[MeSH Subheading] OR “genetics”[All Fields] OR “genetics”[MeSH Terms]) AND (“variant”[All Fields] OR “variant s”[All Fields] OR “variants”[All Fields]) AND (“diagnosable”[All Fields] OR “diagnosi”[All Fields] OR “diagnosis”[MeSH Terms] OR “diagnosis”[All Fields] OR “diagnose”[All Fields] OR “diagnosed”[All Fields] OR “diagnoses”[All Fields] OR “diagnosing”[All Fields] OR “diagnosis”[MeSH Subheading]) AND (“biomarker s”[All Fields] OR “biomarkers”[MeSH Terms] OR “biomarkers”[All Fields] OR “biomarker”[All Fields]) AND (“genome wide association study”[MeSH Terms] OR (“genome wide”[All Fields] AND “association”[All Fields] AND “study”[All Fields]) OR “genome wide association study”[All Fields] OR (“genome”[All Fields] AND “wide”[All Fields] AND “association”[All Fields] AND “studies”[All Fields]) OR “genome wide association studies”[All Fields]).

## 3. Genetic Insights into Endometriosis and Diagnostic Implications

Genome-wide association studies (GWAS) are instrumental in identifying genetic variations associated with diseases. Recent GWAS have provided substantial insights into the genetic architecture of endometriosis, revealing several genetic loci associated with the disease.

### 3.1. Recent Genome-Wide Association Studies (GWAS)

- **Novel Genetic Loci**

Studies have reported multiple new genetic loci associated with endometriosis. For instance, Sapkota et al. [6] identified five novel loci in their meta-analysis (ESR1, CYP19A1, HSD17B1, VEGF and GnRH) which are associated with genes involved in sex steroid regulation and function. A better understanding of these genetic loci could provide potential diagnostic markers for endometriosis.

- **Gene Expression and Pathways**

The genes identified in these loci often play key roles in biological pathways implicated in endometriosis. For example, the genes WNT4 and VEZT have been associated with this disease and are involved in pathways such as hormone regulation and cell adhesion, respectively. Dysregulation of these genes could potentially be used as a diagnostic marker [7,8].

- **Polygenic Risk Scores**

With the accumulation of genetic loci associated with endometriosis from GWAS, there has been increasing interest in developing polygenic risk scores (PRS) [6]. PRS aggregate risk across many genetic variants to predict an individual’s disease risk. Preliminary studies suggest that PRS could be a useful tool in identifying individuals at high risk of developing endometriosis, potentially leading to earlier diagnosis and intervention [7,8].

- **Biomarkers**

The genetic variants identified by GWAS could potentially serve as biomarkers for endometriosis. For example, alterations in the expression of genes associated with endometriosis have been detected in peripheral blood mononuclear cells, suggesting their potential as non-invasive diagnostic markers [8,9].

While these findings offer promising avenues for improving the diagnosis of endometriosis, it is important to note that the genetic basis of endometriosis is complex and still not fully understood. Moreover, the identified genetic variants only explain a small fraction of the disease’s heritability, suggesting that other factors such as rare variants, epigenetic changes, and environmental exposures also play a significant role. Therefore, more comprehensive and integrative studies are needed to fully understand the genetic underpinnings of endometriosis and to translate these findings into diagnostic tools [8,9,10].

### 3.2. Functional Genomics and Endometriosis Diagnosis

Functional genomics has been a game changer in our understanding of complex diseases, including endometriosis. This field of study goes beyond the identification of genetic variants associated with the disease to understand how these variants influence gene function and contribute to disease pathology [9]. Here are some ways in which functional genomics has influenced our understanding of endometriosis and its potential impact on diagnosis.

- **Gene Expression Profiling**

Functional genomic studies often involve gene expression profiling to identify genes that are differentially expressed in endometriotic lesions compared to normal endometrial tissue. For instance, microarray studies have identified several genes that are upregulated or downregulated in endometriosis, which are involved in inflammation, angiogenesis, and extracellular matrix remodeling [10,11]. These differentially expressed genes could potentially serve as diagnostic markers for endometriosis.

- **Epigenetic Modifications**

Epigenetic changes, such as DNA methylation and histone modification, can influence gene expression without altering the DNA sequence. Studies have identified differential methylation patterns in endometriosis, which could influence the disease’s onset and progression. These epigenetic markers could potentially be detected in peripheral blood or endometrial samples, providing non-invasive diagnostic options [8,9,10].

- **Functional Characterization of GWAS Loci**

Functional genomics can help elucidate the biological relevance of the genetic loci identified by GWAS. For example, the fine-mapping and functional annotation of these loci can identify causal variants and target genes. Functional studies can then determine how these variants influence gene function, shedding light on the pathogenesis of endometriosis [10,11].

- **Integration with Other Omics Data**

Functional genomic data can be integrated with other types of omics data, such as proteomics and metabolomics, to provide a more comprehensive understanding of endometriosis. This integrative approach can identify key pathways and molecular signatures of endometriosis, which could be leveraged for diagnosis and targeted therapy [12,13,14].

While functional genomics offer promising avenues for improving the diagnosis of endometriosis, it is important to note that the findings need to be validated in large, independent cohorts. Also, as endometriosis is a heterogeneous disease with diverse clinical presentations, it is crucial to consider this heterogeneity in the design and interpretation of functional genomic studies [15].

### 3.3. Biomarker Discovery and Validation

- **Genetic Biomarkers**

Genetic markers are DNA sequence variations that can be associated with disease susceptibility. In recent years, genome-wide association studies (GWAS) have made significant contributions to identifying genetic biomarkers for endometriosis. These studies involve scanning the entire genome of individuals with and without endometriosis to identify genetic variants that are more common in the affected group [16,17,18].

Important to note is that the identification of genetic biomarkers is just the first step. These markers need further validation in independent cohorts to confirm their association with endometriosis and evaluate their diagnostic performance. Additionally, the functional consequences of these genetic variants and their specific roles in endometriosis pathogenesis need to be elucidated [18,19,20,21].

- **Epigenetic Biomarkers**

Epigenetic modifications, such as DNA methylation, histone modifications, and microRNA expression [22,23], can affect gene expression without altering the DNA sequence. Epigenetic changes have been implicated in endometriosis development and progression, making them potential candidates for biomarker discovery.

Studies have reported different DNA methylation patterns between endometriotic lesions and normal endometria [24]. Lu et al. [25] identified aberrant DNA methylation patterns in genes involved in hormone signaling, inflammation, and cell adhesion in endometriosis. These DNA methylation changes could potentially serve as epigenetic biomarkers for endometriosis diagnosis.

In addition to DNA methylation, microRNAs (miRNAs) have gained attention as potential epigenetic biomarkers for endometriosis. miRNAs are small non-coding RNA molecules that regulate gene expression [25]. Several studies have identified specific miRNAs that are dysregulated in endometriosis tissues compared to normal endometria. These dysregulated miRNAs have the potential to be detected in blood samples, offering a minimally invasive diagnostic approach (Table 1).

- **Biomarker Validation**

Biomarker validation is a crucial step in confirming the diagnostic utility and clinical applicability of the identified markers. Validation involves assessing the performance of biomarkers in independent cohorts, evaluating their sensitivity, specificity, positive and negative predictive values, and determining their diagnostic accuracy [9,24,25].

It’s worth noting that the development of biomarkers for endometriosis is an ongoing process, and further research is needed to refine and validate these potential markers [26]. Large-scale multi-center studies are necessary to validate the diagnostic performance and clinical utility of genetic and epigenetic biomarkers, ultimately leading to their translation into clinical practice for improved endometriosis diagnosis and management.

### 3.4. Technological Advances in Genomics and Diagnostics

New genomic technologies, such as next-generation sequencing (NGS), have revolutionized the field of endometriosis diagnosis by providing more comprehensive and accurate genomic information [27,28]. Here, we provide an explanation of how these technological advances have facilitated advancements in endometriosis diagnosis:

- **Comprehensive Genomic Profiling**

Next-generation sequencing allows for the efficient and high-throughput sequencing of the entire genome or specific target regions, providing a comprehensive view of the genetic landscape. This technology enables the identification of genetic variants associated with endometriosis, including single-nucleotide polymorphisms (SNPs), copy number variations (CNVs), and structural variants. NGS-based approaches have contributed to the discovery of numerous genetic variants linked to endometriosis susceptibility, expanding our understanding of the genetic basis of the disease [28].

- **Identification of Rare Variants**

Endometriosis can have a complex genetic architecture with rare variants playing a role in disease susceptibility. NGS techniques, such as whole exome sequencing (WES) and whole genome sequencing (WGS), enable the identification of rare variants in coding and non-coding regions. By examining the entire coding sequence or the entire genome, these technologies facilitate the discovery of novel genes and pathways associated with endometriosis [26,28,29]. This information enhances our understanding of the molecular mechanisms underlying the disease and provides potential targets for diagnostic purposes.

- **Transcriptomic Analysis**

NGS technologies have greatly advanced transcriptomic analysis in endometriosis. RNA sequencing (RNA-seq) enables the quantification and profiling of gene expression levels in endometriotic lesions and normal endometrial tissues. This approach provides insights into the dysregulation of specific genes and molecular pathways associated with endometriosis. By identifying differentially expressed genes, RNA-seq contributes to the discovery of potential diagnostic biomarkers and improves our understanding of the molecular processes driving endometriosis development and progression [28,30].

- **Epigenetic Profiling**

Epigenetic modifications, such as DNA methylation and histone modifications, play a critical role in gene expression regulation and can be altered in endometriosis. NGS technologies have facilitated comprehensive epigenetic profiling in endometriosis [24,26,29]. Whole genome bisulfite sequencing (WGBS) allows for the mapping of DNA methylation patterns across the genome, enabling the identification of epigenetic changes associated with the disease. Additionally, chromatin immunoprecipitation sequencing (ChIP-seq) provides insights into histone modifications and transcription factor-binding sites, offering a deeper understanding of the epigenetic landscape in endometriosis. These technologies aid in the discovery of potential epigenetic biomarkers for diagnostic purposes [31,32,33].

- **Non-Invasive Diagnostics**

NGS technologies have also facilitated the development of non-invasive diagnostic approaches for endometriosis. Liquid biopsies, which involve the analysis of circulating cell-free DNA or RNA, can be used to detect genetic and epigenetic alterations associated with endometriosis. NGS-based analysis of liquid biopsy samples offers a minimally invasive alternative to traditional diagnostic methods like laparoscopy, providing a more patient-friendly approach [32,33].

In summary, new genomic technologies, particularly next-generation sequencing, have significantly advanced endometriosis diagnosis. These technologies provide a more comprehensive understanding of the genetic and epigenetic landscape of the disease, enable the identification of potential biomarkers, and contribute to our knowledge of the molecular mechanisms underlying endometriosis [23]. As these technologies continue to evolve, they hold promise for further improvements in endometriosis diagnosis and personalized medicine.

## 4. Challenges and Opportunities in Applying Genetic Findings to Endometriosis Diagnosis

Applying genetic findings to endometriosis diagnosis presents both challenges and opportunities. While genetic research has provided valuable insights into the underlying mechanisms of endometriosis [31], translating these findings into clinical practice for improved diagnosis poses several challenges [32]. Here are some of the key challenges and opportunities (Table 2) in applying genetic findings to endometriosis diagnosis.

### 4.1. Challenges

- **Complex Genetic Architecture**

Endometriosis, being a complex, multifactorial disease with a polygenic inheritance pattern, is influenced by multiple genetic variants as well as environmental factors (exposure to environmental toxins, such as dioxin and other endocrine-disrupting chemicals (EDCs), menstrual factors, such as early menarche (start of menstruation), short menstrual cycles, and heavy or prolonged menstrual flow; lifestyle factors, such as alcohol consumption, lack of physical activity, and a diet high in red meat; surgical procedures, particularly those involving the uterus or fallopian tubes; or immune factors, such as alterations in immune response) may play a role in the development of endometriosis [30,32,33].

Unraveling the complex genetic architecture of endometriosis and understanding the interactions between genetic variants poses a significant challenge in applying genetic findings to diagnosis.

- **Genetic Heterogeneity**

Endometriosis exhibits genetic heterogeneity, with different genetic variants being implicated in different populations and ethnic groups. This heterogeneity complicates the development of universal genetic markers or tests that can be applied across diverse populations [33].

- **Low Penetrance of Genetic Variants**

Many of the genetic variants associated with endometriosis have low penetrance, meaning their presence does not guarantee the development of the disease. This poses challenges in using genetic variants as standalone diagnostic markers and highlights the need for incorporating other clinical and environmental factors into diagnostic approaches [33,34].

- **Incomplete Understanding of Genetic Mechanisms**

While genetic studies have identified associations between genetic variants and endometriosis, the precise molecular mechanisms by which these variants contribute to disease development and progression are not yet fully understood. Further research is needed to elucidate these mechanisms, which will enhance the accuracy and clinical applicability of genetic markers in diagnosis [30,34].

### 4.2. Opportunities

- **Identification of Risk Variants**

Genetic findings have identified several risk variants associated with endometriosis.

Most of these have been discovered through genome-wide association studies (GWAS), which search the genome for small variations, called single-nucleotide polymorphisms (SNPs). Here are a few examples of genetic variants that have been associated with endometriosis:

- **Variant on Chromosome 1p36.12**: An SNP in this region was found to be associated with endometriosis in a GWAS [24]. The variant is near the WNT4 gene, which is involved in the development of the female reproductive system.
- **Variant on Chromosome 2p14**: A variant in this region was found to be associated with endometriosis in a study by Nyholt et al. [24]. The nearby gene, GREB1, is involved in estrogen signaling, which plays a role in endometriosis.
- **Variant on Chromosome 7p15.2**: This variant, found near the NFE2L3 gene, was identified in a GWAS by Painter et al. and Nyholt et al. [24]. The function of this gene in endometriosis is not yet known.
- **Variant on Chromosome 9p21**: This variant, found near the CDKN2BAS gene, was identified in a GWAS by Sapkota et al. [6]. This gene is involved in cell cycle regulation.
- **Variant on Chromosome 12q22**: This variant, found near the VEZT gene, was identified in a GWAS by Uimari et al. [10]. VEZT is involved in adherens junctions and cell adhesion.

These variants can serve as potential markers for assessing disease susceptibility and may help identify individuals at higher risk for developing endometriosis. We consider that this information can aid in early detection and preventive strategies [34].

- **Integration of Genetic Markers with Clinical Factors**

Genetic markers can be integrated with clinical factors, such as symptom presentation, imaging results, and biomarkers, to improve diagnostic accuracy. Combining genetic information with clinical data can enhance risk prediction models and contribute to personalized diagnostic approaches [28,30,34].

- **Improved Non-Invasive Diagnostic Methods**

Genetic findings can be utilized to develop non-invasive diagnostic methods, such as liquid biopsies, that rely on the detection of genetic markers in circulating DNA or RNA. These non-invasive approaches offer a patient-friendly alternative to invasive diagnostic procedures and can improve early detection and monitoring of endometriosis.

- **Targeted Therapeutic Approaches**

Understanding the genetic basis of endometriosis can inform targeted therapeutic approaches. Genetic findings may identify specific pathways or genes that can be targeted with novel treatment strategies, leading to more personalized and effective therapies [34].

### 4.3. Anti-Angiogenic Therapy

Angiogenesis, the growth of new blood vessels, is a critical process in the development and maintenance of endometriotic lesions. Therapies that inhibit angiogenesis are being investigated as potential treatments for endometriosis. Drugs such as bevacizumab, an antibody that inhibits vascular endothelial growth factor (VEGF), a key molecule in angiogenesis, are currently being tested in clinical trials [23,26].

### 4.4. Immunotherapy

Research has shown that the immune system plays a significant role in the development of endometriosis. Therapies that modulate the immune response, such as the use of anti-TNF alpha drugs, are being explored [28].

### 4.5. Hormonal Therapy

Newer hormonal treatments are being developed that target specific pathways involved in endometriosis. For example, selective progesterone receptor modulators (SPRMs) and aromatase inhibitors are being investigated for their ability to suppress the growth of endometrial tissue [19,28].

Despite the challenges, ongoing research and advancements in genetic technologies hold promise for improving endometriosis diagnosis. Integrating genetic findings with other clinical and environmental factors, along with advancements in non-invasive diagnostic approaches, will likely enhance our ability to accurately diagnose endometriosis and provide tailored treatments to individuals affected by the disease [32].

## 5. Emerging Trends and Future Directions

Genetic research in endometriosis is continuously evolving, and emerging trends are shaping future diagnostic techniques. Two prominent emerging trends in genetic research that could have a significant impact on future diagnostic techniques in endometriosis are polygenic risk scores (PRS) and multi-omics approaches. In the ensuing section, we would like to discuss each of these trends in more detail [33,34].

### 5.1. Polygenic Risk Scores (PRS)

Polygenic risk scores involve the calculation of a cumulative genetic risk score based on multiple genetic variants associated with a particular condition. In endometriosis, researchers are developing polygenic risk scores by considering the combined effects of numerous genetic variants that contribute to disease susceptibility [31,32]. These scores can estimate an individual’s genetic predisposition to endometriosis and provide a personalized risk assessment [34].

The use of polygenic risk scores in endometriosis diagnosis has several potential benefits. PRS can improve risk prediction and identify individuals at higher risk for developing endometriosis. They can also help stratify patients based on disease severity or response to treatment, guiding personalized management approaches. Additionally, polygenic risk scores may aid in distinguishing between different endometriosis subtypes or disease phenotypes, enhancing diagnostic precision [34].

### 5.2. Multi-Omics Approaches

Multi-omics approaches integrate multiple layers of molecular information, such as genomics, transcriptomics, epigenomics, proteomics, and metabolomics, to gain a comprehensive understanding of disease mechanisms. In endometriosis, multi-omics studies are shedding light on the complex molecular alterations associated with the condition, providing insights into potential diagnostic biomarkers and therapeutic targets [25].

By combining genomic data with other omics data, such as transcriptomic profiles or epigenetic modifications, researchers can identify key molecular pathways and networks involved in endometriosis. These multi-omics approaches may unveil novel diagnostic markers that can improve the accuracy and specificity of endometriosis diagnosis. They can also enhance our understanding of the disease’s underlying mechanisms and guide the development of targeted therapies.

The integration of polygenic risk scores and multi-omics approaches has the potential to transform endometriosis diagnosis by providing a more comprehensive and personalized assessment. By considering the cumulative genetic risk and incorporating multi-dimensional molecular data, clinicians can improve risk prediction, diagnostic accuracy, and treatment decision making for individuals with endometriosis [31,32,33,34].

### 5.3. The Importance of Genetic Mutations in the Malignant Transformation of Endometriosis

Lately, studies have focused on highlighting the importance of genetic mutations in the possible malignant transformation of endometriosis, with findings indicating a correlation between mutations in CTNNB1-encoding B-catenin [35] levels in this regard. Another genetic mutation involved in this process would be the tumor suppressor gene ARID1A (A-rich interactive domain-containing protein 1A) [35,36,37]. Involvement in the loss of heterozygosity and other mutations with possible associations include PTEN loss, JAZF1-SuZ12, and EPC1-PHF1, which have also been studied, but a clear conclusion requires extensive research [37,38].

However, it is important to note that these emerging trends are still in the early stages of development, and further research is needed to validate their clinical utility and assess their real-world impact. Large-scale collaborative efforts, rigorous validation studies, and integration with clinical parameters are necessary to translate these trends into practical diagnostic techniques that can be implemented in routine clinical practice. This is also significant because, thus far, none of the studied biomarkers, both genetic and of other nature, such as the extensively studied CA 125, have proven their clear utility in non-invasive and accurate diagnosis of endometriosis, without providing the possibility of avoiding surgical intervention and histopathological examination as the gold standard diagnostic [39].

### 5.4. The Applicability of Genetics in Establishing the Diagnosis of Endometriosis in Adolescents

Genetic research is exploring the role of genetics in diagnosing endometriosis in adolescents. Although laparoscopy is the definitive diagnostic procedure, genetic studies provide insights into underlying mechanisms and potential genetic markers [17,40]. Key findings include the following.

- **Familial aggregation**

Endometriosis often runs in families, indicating a genetic component. First-degree relatives have a higher risk, suggesting genetic factors contribute to susceptibility [40].

- **Candidate gene studies**

Genes involved in hormonal regulation, inflammation, and tissue remodeling, such as estrogen receptors (ESR1, ESR2), progesterone receptors (PGRs), and immune-related genes (TNF-α, IL-1β), are investigated. Variants in these genes may influence endometriosis risk [40,41].

- **Genome-wide association studies (GWAS)**

Common genetic variants associated with endometriosis are identified, providing insights into the condition’s genetic architecture and potential susceptibility regions [40,41].

- **Epigenetic alterations**

Modifications like DNA methylation and non-coding RNA expression can influence gene expression patterns, possibly impacting endometriosis development. Understanding the epigenetic landscape may aid in diagnostic marker identification.

However, it is important to note that genetic studies are still in early stages, and more research is needed. Genetic testing alone cannot definitively diagnose endometriosis. Clinical evaluation, symptom assessment, and imaging studies (ultrasound, MRI) remain crucial in diagnosing adolescents [17,40,41].

### 5.5. The Financial Barrier between Diagnostic Means and Clinical Practice

Integrating genetic testing, like multi-gene panels, for diagnosing endometriosis in adolescents faces financial challenges, limiting widespread accessibility [17,40,41]. Key financial considerations include the following.

- **Cost**

Genetic testing, especially multi-gene panels, can be expensive. It includes testing, analysis, interpretation, and reporting. The panel’s complexity and number of genes impact the overall cost [41].

- **Insurance Coverage**

Coverage varies by country, region, and policies. Some insurance plans cover endometriosis genetic testing, while others have limited coverage or specific criteria. Lack of coverage or high deductibles create financial barriers [41].

- **Limited Reimbursement**

Even with insurance coverage, reimbursement rates may not fully cover testing costs. This burdens patients with remaining balances and may lead to disparities based on socioeconomic factors [41].

- **Availability of Genetic Counselors**

Genetic counseling, important before and after testing, may be limited in certain areas. Limited access to comprehensive counseling exacerbates financial barriers [41].

- **Research and Development Costs**

Developing and validating multi-gene panels require significant investment. These costs contribute to the higher price, especially for newer and comprehensive panels.

Addressing these barriers requires collaboration among healthcare providers, policymakers, and researchers. Balancing the benefits of genetic testing for endometriosis with financial considerations is crucial. Minimizing patient financial burden and promoting equitable access should be prioritized [41].

## 6. Discussion

This comprehensive review on genetic insights into endometriosis and their diagnostic implications reveals several key findings. Firstly, genetic research has identified numerous genetic variants associated with endometriosis, shedding light on the complex genetic architecture of the disease. These findings have the potential to improve our understanding of the underlying molecular mechanisms and pathways involved in endometriosis development. It also emphasizes the significance of genetic contributions in improving endometriosis diagnosis. Genetic insights offer the potential for more accurate and early detection of endometriosis, allowing for timely intervention and personalized treatment strategies [1,3,7,8].

The identification of genetic markers, such as single-nucleotide polymorphisms (SNPs), copy number variations (CNVs), and epigenetic modifications, holds promise for the development of diagnostic tests and risk prediction models. Furthermore, the integration of genetic information with clinical factors, imaging findings, and biomarkers can enhance the precision and reliability of endometriosis diagnosis. This holistic approach has the potential to improve diagnostic accuracy, guide treatment decisions, and monitor disease progression [10,15,20].

We set out to highlight the future directions for research in the field of endometriosis genetics, but further validation and replication studies are needed to confirm the clinical utility of genetic markers and their potential translation into routine clinical practice. Large-scale multi-center collaborations are essential, as well, to overcome the challenges of genetic heterogeneity and ensure the generalizability of diagnostic tools across diverse populations [27].

In addition, this review suggests the exploration of emerging technologies and approaches, such as polygenic risk scores and multi-omics integration, to advance endometriosis diagnosis. Polygenic risk scores can provide personalized risk assessments, while multi-omics approaches can unveil novel diagnostic biomarkers and therapeutic targets. Continued research into the genetic basis of endometriosis and the integration of genetic findings with other omics data and clinical parameters are crucial for advancing diagnostic techniques in the field. Although this research may be expensive, it is necessary to improve the accuracy and effectiveness of diagnostic methods. Currently, it may not be cost-effective, as indicated by studies [28,29,30,32,34,41]. However, by investing in further research and development, there is potential to enhance the cost-effectiveness of these diagnostic approaches in the future.

## 7. Conclusions

Within this review, we proposed to highlight the transformative potential of genetic insights in endometriosis diagnosis. Genetic markers, risk prediction models, and multi-dimensional approaches have the capacity to revolutionize the field by enabling earlier detection, accurate risk assessment, and personalized management strategies [34]. Likewise, future research should focus on the validation, replication, and the integration of genetic findings into routine clinical practice to maximize the clinical utility of genetic insights in endometriosis diagnosis and patient care.

## Figures and Tables

**Table 1:** Key biomarkers discovered and validated for endometriosis.

Biomarker	Type	Diagnostic Potential	Validation Findings	References
CA-125	Protein	Potential biomarker for endometriosis diagnosis	Elevated levels in women with endometriosis compared to controls	[9]
miRNA-200 family	MicroRNA	Potential diagnostic biomarkers for endometriosis	Differential expression in endometriosis compared to controls	[17]
miR-200b	MicroRNA	Promising diagnostic biomarker for endometriosis	Increased expression in endometriosis compared to controls	[17]
HE4 (Human Epididymis Protein 4)	Protein	Potential diagnostic marker for endometriosis	Elevated levels in endometriosis compared to controls	[19]
Circulating cell-free DNA	Nucleic Acid	Potential biomarker for endometriosis diagnosis	Altered DNA methylation patterns in endometriosis	[25]

**Table 2:** The challenges faced in understanding and diagnosing endometriosis while presenting the corresponding opportunities offered by genetic insights.

Challenges	Opportunities
Elusive understanding of endometriosis pathogenesis	Genetic insights shedding light on molecular mechanisms
Lack of non-invasive diagnostic methods	Integration of genetic markers for improved diagnosis
Heterogeneous clinical presentation	Personalized treatment strategies based on genetic insights
Delayed diagnosis or misdiagnosis	Early and accurate diagnosis through genetic markers
Validation of genetic markers and risk prediction models	Enhanced risk assessment and stratification
Translating genetic findings into routine clinical practice	Incorporation of genetic information into diagnostics
Ethical considerations	Ethical guidelines for responsible genetic testing
Implementation challenges in clinical settings	Adoption of genetic insights into clinical practice
Long-term monitoring and disease management	Identification of markers for disease progression

## Data Availability

Not applicable.
